# Air Quality Perceptions, Awareness, and Associated Behaviors Among U.S. Adults With and Without Heart Disease

**DOI:** 10.1016/j.focus.2024.100249

**Published:** 2024-06-06

**Authors:** Tia C. Dowling, Audrey F. Pennington, Hilary K. Wall, Maria C. Mirabelli

**Affiliations:** 1 Asthma and Air Quality Branch, Division of Environmental Health Science and Practice, National Center for Environmental Health, Centers for Disease Control and Prevention, Atlanta, Georgia; 2 Oak Ridge Institute for Science and Education, Oak Ridge, Tennessee; 3 Lead Poisoning Prevention and Surveillance Branch, Division of Environmental Health Science and Practice, National Center for Environmental Health, Centers for Disease Control and Prevention, Atlanta, Georgia; 4 Division for Heart Disease and Stroke Prevention, National Center for Chronic Disease Prevention and Health Promotion, Centers for Disease Control and Prevention, Atlanta, Georgia

**Keywords:** Air pollution, epidemiology, heart disease, prevalence, survey

## Abstract

**Introduction:**

Exposure to ambient air pollution can worsen cardiovascular disease and increase the risk of stroke, myocardial infarction, and cardiovascular disease mortality. Strategies to reduce air pollution exposure can therefore help prevent cardiovascular morbidity and mortality. This study was conducted to assess the awareness among U.S. adults of the effect of air pollution on cardiovascular health and actions individuals can take to reduce their air pollution exposure.

**Methods:**

In May–July 2022, 4,156 adults responded to the summer wave of the 2022 ConsumerStyles survey and self-reported their heart disease status and perceptions, awareness, and behaviors about ambient air pollution and health. In 2023, the data were analyzed to generate weighted population estimates representative of noninstitutionalized U.S. adults. Associations between heart disease and responses about perceptions, awareness, and behaviors were estimated using binomial and multinomial regression methods for weighted data.

**Results:**

Overall, 90% of the weighted population estimate of U.S. adults reported that air pollution can impact a person's health, and 44% reported that air pollution can cause or worsen heart disease. Percentages of adults reporting that air pollution can impact a person's health (prevalence ratio=1.09; 95% CI=1.06, 1.12) and that air pollution can cause or worsen heart disease (prevalence ratio=1.28; 95% CI=1.08, 1.51) were higher among adults with than without heart disease.

**Conclusions:**

Less than half of U.S. adults are aware that air pollution affects heart disease. Improvements in awareness of the effect of air pollution on cardiovascular health and strategies to reduce exposure could help protect individuals with heart disease.

## HIGHLIGHTS

• Exposure to ambient air pollution can worsen heart disease
• Less than half of U.S. adults are aware that air pollution affects heart disease
• Awareness that air pollution affects health is higher in adults with heart disease
• Awareness of actions to reduce exposure could protect people with heart disease

## INTRODUCTION

Exposure to ambient air pollution is a risk factor for the onset or exacerbation of many health conditions, including cardiovascular disease, diabetes, lung cancer, obesity, respiratory disease, and a shortened life expectancy.^1^^,^^2^ Associations between cardiovascular disease and related hospitalizations and exposure to air pollutants, including particulate matter less than or equal to 2.5 micrometers in aerodynamic diameter (PM_2.5_), carbon monoxide, nitrogen dioxide, and sulfur dioxide exposure are well described.^2^^,^^3^ Global estimates suggest that air pollution is responsible for nearly 20% of all cardiovascular disease.^4^

Common sources of ambient air pollution in the U.S. include motor vehicles, forest fires, and factories.^5^ Ambient air pollution is inhaled into the body and enters the lungs and blood stream, disrupting many of the body systems. Exposure to air pollution can lead to physiological changes in the heart, such as the buildup of plaque and an increased risk of heart attack, stroke, and related hospitalizations.^2^^,^^4^^,^^6^ As some policy-based strategies to reduce ambient air pollution can take years to implement and improve air quality, individual-based exposure reduction behaviors are also important to minimize exposure-based health effects.^7^ Given the well-described risk of cardiovascular health outcomes because of air pollution, any behavior that reduces air pollution exposure is expected to reduce negative health outcomes.^7^ Examples of changes people can make to help minimize their air pollution exposure on days with poor air quality include spending less time outdoors, doing less strenuous activities outside, or avoiding being outside at certain times of the day. However, for people to intentionally take actions that can effectively reduce their air pollution exposure, they must be aware of both the air quality in their environment and the actions that can lead to reductions in exposure.

A previous study that used similar methods to this study found moderate levels of awareness of air quality alerts among U.S. adults, yet behavior modifications remain relatively uncommon. An analysis of survey data from 2016 to 2018 reported that although 54% of respondents were aware of air quality alerts, only 29% reported thinking or were informed that air quality was bad, and just 15% of those reported changing their behavior as a result.^8^ The study found no statistically significant difference in air quality awareness among adults with and without heart disease. These results indicate low awareness of air pollution and an opportunity for improving awareness about strategies to reduce the burden of cardiovascular disease, including exacerbations of existing heart disease. Million Hearts, a national initiative co-led by the Centers for Disease Control and Prevention (CDC) and the Centers for Medicare and Medicaid Services with the goal of preventing acute cardiovascular events, is focusing on decreasing particle pollution exposure by raising awareness of its impact on heart disease and changing related behaviors.^9^ This study was conducted among U.S. adults surveyed May 31–July 6, 2022 to assess awareness of the effect of air pollution on cardiovascular health and actions individuals can take to reduce their air pollution exposure.

## METHODS

### Study Sample

This study was conducted using responses to the summer wave of the 2022 ConsumerStyles survey. ConsumerStyles surveys are conducted by Porter Novelli Public Services (Washington, DC) as cross-sectional surveys in the spring, summer, and fall each year. The spring wave of the survey (hereafter referred to as SpringStyles) was conducted between March 22 and April 15, 2022, and sent to 11,390 potential respondents. Potential respondents were randomly selected using probability-based sampling from KnowledgePanel, a panel of approximately 60,000 noninstitutionalized adults aged 18 years and older. The SpringStyles survey was completed by 6,601 adults, yielding a response rate of 58.0%. Between May 31 and July 6, 2022, the summer wave of the survey (hereafter referred to as *SummerStyles*) was sent to 5,990 adults who had completed the SpringStyles survey. The SummerStyles survey was completed by 4,156 respondents, yielding a response rate of 69.4%.

### Measures

Survey respondents self-reported demographic information and health conditions. Health conditions were asked in a multi-option question specifically about disease currently or within the last year. Respondents who reported *atrial fibrillation, congestive heart failure, or other heart condition (angina or heart attack)* were identified as having a history of heart disease. All remaining respondents were categorized as having no history of heart disease.

Participants were asked a series of questions to assess their perceptions, awareness, and behaviors about ambient air pollution. The survey questions began with *The next few questions are about air pollution.* Respondents then answered a series of questions about their perceptions about the relationship between air pollution and health, awareness of air quality alerts, behavior changes they make because of poor air quality, and discussions with healthcare professionals (HCPs) about strategies to reduce air pollution exposure. As in previous analyses of ConsumerStyles data, all negative (i.e., no) and uninformative (i.e., don't know, missing) responses were categorized as negative.^10^

### Statistical Analysis

ConsumerStyles data were weighted to correspond to the U.S. Current Population Survey proportions of the following factors: sex by age, household income, race/ethnicity, household size, education, census region, metropolitan statistical area (MSA) status, and parental status of children 12–17 years old. All analyses were performed in 2023 and were weighted using the survey weights provided by Porter Novelli Public Services; the results represent the weighted population estimate of noninstitutionalized U.S. adults aged 18 years and older.

Weighted percentages and 95% CIs were generated for affirmative responses to each survey question, and prevalence ratios (PRs) with 95% CIs were generated using logistic binomial or multinomial regression to assess whether responses varied by heart disease status. Frequencies and unadjusted weighted percentages with 95% CIs were generated using R 4.2.2.^11^ Adjusted weighted percentages with 95% CIs and PRs with 95% CIs were calculated using predicted marginal probabilities from logistic regression models in SAS-callable SUDAAN (RTI International, Research Triangle Park, North Carolina).^12^ For questions that contained multiple response options, calculations were made for each option. The regression models were adjusted for age (18–29, 30–44, 45–59, 60–74, ≥75 years), educational attainment (less than high school, high school, some college, bachelor's degree or higher), MSA status (metropolitan, nonmetropolitan), race and ethnicity (non-Hispanic White; non-Hispanic Black; non-Hispanic other; Hispanic; ≥2 races, non-Hispanic), U.S. Census region (northeast, midwest, south, west), and sex (female, male). Two sensitivity analyses were performed to assess the impact of comorbid chronic conditions based on the results of the main analysis. The first sensitivity analysis used the same model as the main analysis but excluded 432 respondents with self-reported asthma, emphysema, or chronic obstructive pulmonary disease (COPD). The second sensitivity analysis used the same model as the main analysis but excluded 1,476 respondents with self-reported asthma, emphysema, COPD, or seasonal allergies. This activity was reviewed by the CDC, deemed exempt from IRB review, and conducted consistent with applicable federal law and CDC policy (see e.g., 45 C.F.R. part 46; 21 C.F.R. part 56; 42 U.S.C. §241(d), 5 U.S.C. §552a, 44 U.S.C. §3501 et seq).

## RESULTS

Demographic characteristics are shown in Table 1. The average age of respondents was 55.3 years (SD=16.5 years). In the surveyed population, 177 respondents, corresponding to 3.7% (95% CI=3.1, 5.3) of the weighted population estimate, self-reported a history of heart disease.

**Table 1:** Demographic Characteristics of 2022 SummerStyles Respondents

Respondent characteristics	n^a^	Weighted % (95% CI)
Age, years		
18–29	364	19.1 (17.3, 20.9)
30–44	1,034	26.7 (25.0, 28.3)
45–59	1,094	22.9 (21.4, 24.3)
60–74	1,235	23.8 (22.4, 25.1)
≥75	429	7.6 (6.9, 8.4)
Educational attainment		
Less than high school	216	9.5 (8.2, 10.9)
High school	1,036	28.3 (26.6, 30.0)
Some college	1,167	27.1 (25.5, 28.7)
Bachelor's degree or higher	1,737	35.1 (33.5, 36.8)
Metropolitan Statistical Area (MSA) status		
Metropolitan area	3,626	86.7 (85.4, 87.9)
Nonmetropolitan area	530	13.3 (12.1, 14.6)
Race/ethnicity		
Non-Hispanic White	2,981	62.7 (60.8, 64.6)
Non-Hispanic Black	382	11.9 (10.6, 13.2)
Other, non-Hispanic	205	7.2 (6.1, 8.3)
Hispanic	467	16.8 (15.2, 18.4)
2+ races, non-Hispanic	121	1.5 (1.1, 1.8)
U.S. Census region^b^		
Midwest	925	20.6 (19.2, 22.1)
Northeast	741	17.2 (15.9, 18.6)
South	1,497	38.2 (36.4, 40.0)
West	993	23.9 (22.3, 25.5)
Sex		
Female	2,081	51.1 (49.3, 53.0)
Male	2,075	48.9 (47.0, 50.7)

a Unweighted frequency.

b Defined by the U.S. Census Bureau: Northwest: Connecticut, Massachusetts, Maine, New Hampshire, New Jersey, New York, Pennsylvania, Rhode Island, Vermont; Midwest: Iowa, Illinois, Indiana, Kansas, Michigan, Minnesota, Michigan, North Dakota, Nebraska, Ohio, South Dakota, Wisconsin; South: Alabama, Arkansas, Delaware, Florida, Georgia, Kentucky, Louisiana, Maryland, Missouri, North Carolina, Oklahoma, South Carolina, Tennessee, Texas, Virginia, West Virginia, Washington DC; West: Alaska, Arizona, California, Colorado, Hawaii, Idaho, Montana, New Mexico, Nevada, Oregon, Utah, Washington, Wyoming.^13^

Overall, 89.8% of the weighted population estimate reported thinking that air pollution can impact a person's health (Table 2). The weighted estimate was higher among persons with heart disease (97.7%) than among persons without heart disease (89.5%) (adjusted PR=1.09; 95% CI=1.06, 1.12) (Table 2). Despite the high prevalence of awareness that air pollution affects health, only 58.7% of adults reported thinking that there are things they can do to limit their or their family's exposure to air pollution, and the percentages among adults with and without heart disease were similar (Table 2). Among adults who recognized that air pollution impacts health, 44.2% reported thinking that heart disease can be caused or worsened by air pollution (Table 2). This percentage was notably higher among adults with than without heart disease (adjusted PR=1.28; 95% CI=1.08, 1.51) (Table 2).

**Table 2:** Air Quality Perceptions, Awareness, and Behaviors among U.S. Adults and Associations with Heart Disease Status

Survey questions	All respondents (n= 4,156)		With heart disease (n=177)		Without heart disease (n= 3,946)			
	n^a^	Weighted % (95% CI)	n^a^	Weighted % (95% CI)	n^a^	Weighted % (95% CI)	Unadjusted PR	Adjusted PR^b^
Do you think air pollution can impact a person's health in any way?	3,816	89.8 (88.6, 91.1)	173	97.7 (95.5, 100)	3,643	89.5 (88.2, 90.8)	1.09 (1.06, 1.12)	1.09 (1.06, 1.12)
Do you think there are things you can do to limit your or your family's exposure to air pollution?	2,540	58.7 (56.9, 60.5)	105	55.3 (46.9, 63.7)	2,435	58.8 (56.9, 60.7)	0.94 (0.81, 1.10)	0.96 (0.82, 1.12)
Do you think heart disease can be caused or worsened by air pollution?^c^	1,756	44.2 (42.4, 46.1)	107	58.2 (49.7, 66.7)	1,649	43.7 (41.7, 45.6)	1.33 (1.14, 1.55)	1.28 (1.08, 1.51)
Have you ever heard or read about the Air Quality Index or air quality alerts where you live?	2,499	54.7 (52.8, 56.5)	115	60.8 (52.5, 69.1)	2,384	54.4 (52.5, 56.3)	1.12 (0.97, 1.29)	1.05 (0.90, 1.24)
During the past 12 months, was there any time you thought or you were informed that air quality where you live was bad?	1,620	36.5 (34.7, 38.2)	79	41.2 (33.1, 49.3)	1,541	36.3 (34.5, 38.1)	1.13 (0.93, 1.39)	1.20 (0.97, 1.48)
Did you do anything differently when you thought or were informed that air quality where you live was bad?^d^	909	57.2 (54.3, 60.1)	36	45.8 (33.2, 58.3)	873	57.7 (54.7, 60.7)	0.79 (0.60, 1.05)	0.88 (0.67, 1.15)
Which of these did you do differently when you thought air quality was bad?^e^								
Spent less time outdoors	819	90.3 (88.0, 92.7)	28	85.5 (74.9, 96.1)	791	90.5 (88.1, 92.9)	0.95 (0.83, 1.07)	0.90 (0.75, 1.08)
Did less strenuous activities	260	27.0 (23.6, 30.4)	13	30.0 (14.0, 46.0)	247	26.9 (23.4, 30.3)	1.12 (0.65, 1.94)	0.87 (0.45, 1.67)
Closed windows of house	592	65.7 (62.0, 69.4)	25	62.3 (42.2, 82.4)	567	65.8 (62.0, 69.5)	0.95 (0.68, 1.31)	0.93 (0.69, 1.27)
Drove my car less	174	19.1 (16.0, 22.2)	8	21.0 (6.1, 35.9)	166	19.0 (15.8, 22.2)	1.10 (0.53, 2.29)	0.94 (0.46, 1.94)
Exercised indoors instead of outdoors	305	30.0 (26.5, 33.4)	12	31.5 (14.0, 48.9)	293	29.9 (26.4, 33.5)	1.05 (0.60, 1.85)	0.92 (0.51, 1.65)
Exercised on a different day/different time	148	15.6 (12.8, 18.3)	6	11.2 (2.1, 20.2)	142	15.7 (12.9, 18.5)	0.71 (0.31, 1.63)	0.74 (0.31, 1.75)
When walking, biking, or exercising outdoors, how often do you avoid busy roads to reduce your exposure to air pollution?								
Always	583	14.0 (12.8, 15.3)	27	13.9 (8.6, 19.1)	556	14.0 (12.7, 15.3)	0.99 (0.67, 1.46)	0.86 (0.56, 1.32)
Usually	810	18.2 (16.8, 19.6)	42	21.8 (15.2, 28.3)	768	18.1 (16.7, 19.5)	1.20 (0.88, 1.64)	1.26 (0.92, 1.73)
Sometimes	763	19.2 (17.7, 20.7)	20	11.4 (5.9, 16.9)	743	19.5 (18.0, 21.0)	0.58 (0.36, 0.96)	0.57 (0.34, 0.94)
Rarely	714	17.2 (15.8, 18.6)	21	11.1 (6.2, 16.0)	693	17.4 (16.0, 18.9)	0.64 (0.41, 1.00)	0.82 (0.51, 1.32)
Never	1,286	31.3 (29.6, 33.0)	67	41.8 (33.4, 50.2)	1,219	30.9 (29.2, 32.7)	1.35 (1.10, 1.66)	1.30 (1.02, 1.66)
Have you and your doctor, nurse practitioner, or other health care professional ever talked about how to limit your exposure to air pollution?	138	3.3 (2.7, 4.0)	6	3.0 (0.5, 5.4)	132	3.4 (2.7, 4.0)	0.88 (0.38, 2.06)	0.44 (0.15, 1.32)

a Unweighted frequency of affirmative responses.

b Adjusted for age, educational attainment, MSA status, race/ethnicity, U.S. Census region, and sex.

c Among the 3,816 respondents who reported thinking that air pollution can impact a person's health in any way.

d Among the 1,620 respondents who reported thinking or being informed in the past 12 months that air quality where they live was bad.

e Among the 909 respondents who reported doing anything differently when they thought or were informed that air quality where they live was bad. PR, prevalence ratio.

An estimated 54.7% of U.S. adults reported having heard or read about air quality alerts where they live, and 36.5% thought or were informed that air quality was bad where they lived during the past 12 months (Table 2). In adjusted models, percentages of respondents reporting having heard or read about air quality alerts and thinking or being informed that air quality where they lived was bad during the past 12 months were both higher among adults with heart disease than among adults without heart disease, though the 95% CIs included unity (PR=1.05; 95% CI=0.90, 1.24, PR=1.20; 95% CI=0.97, 1.48, respectively) (Table 2). Of the estimated 36.5% of U.S. adults who reported thinking or being informed that air quality was bad in the past 12 months, 57.2% reported doing something differently in response to this information.

Among adults who reported changing their behaviors when they thought or were informed that air quality was bad, the most common actions reported were spending less time outdoors (90.3%) and closing windows in the house (65.7%) (Table 2); 32.2% reported always or usually avoiding busy roads to reduce exposure to air pollution, whereas only 3.3% reported talking with an HCP about how to limit their exposure to air pollution. Overall, heart disease status was not associated with spending less time outdoors, closing windows in the house, or other exposure-reducing behaviors.

A comparison of the characteristics of adults who responded to the survey and adults who did not revealed similarities in demographics and socioeconomics, with a notable exception. The average age of respondents (53.3 years) was higher than the average age of nonrespondents (47.3 years). Although a chi-squared test did not indicate statistically significant differences across age groups, the population of respondents included a higher proportion of individuals aged 60 years and older (40.0%) than the population of nonrespondents (15.4%), indicating a notably higher participation among older adults.

The results of the 2 sensitivity analyses were consistent with the results of the main analysis in magnitude and precision. For example, the adjusted PR for thinking that air pollution can impact a person's health for the main analysis was 1.09 (95% CI=1.06, 1.12) compared with 1.08 (95% CI=1.05, 1.12) and 1.09 (95% CI=1.04, 1.15) for the first and second sensitivity analyses, respectively.

## DISCUSSION

Overall, these data show that a high percentage of U.S. adults are aware of the impact of air pollution on health, and that air quality awareness is generally higher among adults with than without heart disease. However, with less than half of adults believing that heart disease can be caused or worsened by air pollution and with more than a third of adults unaware of the actions they can take to reduce their or their family's exposure to air pollution, these findings also reveal opportunities for improvements in awareness of the effect of air pollution on cardiovascular health and about strategies to reduce exposure to ambient air pollution.

These results support and extend findings from 2 previous analyses of ConsumerStyles data. In 2018, Mirabelli et al.^8^ reported on air quality awareness among U.S. adults with and without respiratory disease and heart disease. In 2020, Mirabelli et al.^14^ extended those findings by reporting on associations between the Air Quality Index and air quality awareness and showed that selected aspects of air quality awareness increased across categories of increased numbers of days with Air Quality Index values ≥101, a value corresponding to a categorization of *unhealthy for sensitive groups*. This study reports on an analysis of 2022 ConsumerStyles data, focused on U.S. adults with and without heart disease, and extends the findings of both previous studies by reporting on a wider range of topics related to air quality perceptions, awareness, and associated behaviors. As a result, findings from this study can, in part, be compared with results from the previous studies. For example, 49% of U.S. adults in 2014–2016 and 54% of U.S. adults in 2016–2018, compared with 55% in this analysis, were aware of air quality alerts where they live.^8^^,^^10^ Neither the analysis of 2014–2016 data nor the analysis of 2016–2018 data reported a difference in awareness of air quality alerts by heart disease status. In this study, although the percentage of adults with heart disease reporting awareness of air quality alerts (61%) was higher than the percentage of adults with heart disease reported in the previous studies (49% in 2014–2016; 53% in 2016–2018), and the adjusted point estimate of the association was higher and farther from the null, the 95% CIs of the adjusted estimates in all 3 studies included unity.^8^^,^^10^ Previous research has shown 2 factors that influence air quality awareness. Results from a media campaign on air pollution found that perception of personal risk greatly influences information-seeking behavior, with those with respiratory disease more likely to seek out air quality information than individuals with cardiovascular disease.^15^ Another study found increased air quality awareness in areas with a reported Air Quality Index, even in areas where the Air Quality Index did not exceed a categorization of moderate.^8^ However, a study from 2008 found that air quality alerts were not sufficient to cause behavior change.^16^ Rather, adults were motivated to change their behavior by their own perception of air quality.^16^ Together, these studies indicate a gap between air quality awareness and changes in behaviors to reduce air pollution exposure. Interventions designed to address this gap might be strengthened by the incorporation of efforts not only to increase air pollution awareness but also to identify and address changes in behaviors to reduce air pollution exposure. Following Air Aware, a local intervention conducted in Pittsburgh, Pennsylvania, that was designed to promote access to air quality forecasts and promote self-protective behaviors related to air quality, investigators reported that research-based communication with populations at increased risk of air quality–related exacerbations of their health can improve prosocial behaviors during times of poor air quality.^15^ One strategy could be to strengthen the public's understanding and use of air quality alerts, which are the main tool for communicating health risks from air pollution in the U.S. Given the hourly fluctuations in air quality, hourly reporting provides real-time information that individuals can use to adjust their activity levels throughout the day to best limit air pollution exposure.

Across all the ConsumerStyles analyses discussed, changing behavior in response to awareness of air quality was uncommon. The study of 2016–2018 data reported that 15% of all U.S. adults and 16% of U.S. adults with heart disease changed their behaviors when they were aware of poor air quality.^8^ In this study, responses to the same survey question were assessed among only adults who reported thinking or being informed of poor air quality, resulting in a finding that an estimated 57% of U.S. adults changed their behavior when they thought or were informed that air quality was poor. When these same responses were assessed among all adults to compare them with the previous results, an estimated 21% of all U.S. adults and 19% of U.S. adults with heart disease reported changing their behaviors when they were aware of poor air quality. In 2014, Roberts et al.^17^ reported that increased concentrations of PM_2.5_ were associated with an increase in physical inactivity. Together, these results suggest opportunities to improve awareness about strategies to reduce one's exposure to air pollution when air quality is poor.

Analysis of survey data from 2014–2016 found that 3% of U.S. adults have discussed strategies to limit air pollution exposure with an HCP.^10^ This study reports similar estimates (3.3%). Conversely, an analysis of survey data from 2015, which used similar survey methods but focused on HCPs, found that 41% of HCPs reported having ever talked to patients about limiting air pollution exposure.^18^ Together, these studies indicate an opportunity for improving patient–provider discussions about air pollution and its associated health effects.

Education-focused interventions to address the gaps in air quality awareness identified in this study and additional information about the self-efficacy of these interventions have the potential to notably improve the general understanding of air pollution and health among the public, including among patients with cardiovascular disease and other health conditions that put them at an increased risk for negative health outcomes.

### Limitations

There are some potential limitations of this study to consider. Information collected in the ConsumerStyles survey, including information about demographic characteristics and health status, was self-reported and not validated. Such methods introduce the potential for the results presented in this study to be affected by misclassification because of misreporting. Misreporting can occur for behavior-based questions because of recall bias.^19^ The survey questions ask about air pollution without delineating between ambient and indoor air pollution. Respondents may have considered indoor air pollution in their responses, whereas this analysis focuses on ambient air pollution. The survey question regarding health status focused solely on information from the past year. As such, respondents may have had heart-related events predating this period yet were classified as not having a history of heart disease based on these inclusion criteria. Because of the low numbers of respondents with self-reported heart disease, stratified analyses could not be performed.

## CONCLUSIONS

As air pollution exposure is a modifiable risk factor for cardiovascular disease, exposure reduction actions, such as spending less time outdoors, doing less strenuous activities outside, or avoiding being outside at certain times of day, can be performed by individuals to decrease their exposure. Individuals need to be aware of the role of air pollution in cardiovascular disease, air quality levels, and effective exposure reduction behaviors. Although 89.8% of the population believes that air pollution can affect health, only 44.2% think that air pollution could cause or worsen heart disease. Among the individuals who were aware of poor air quality alerts, 57.2% reported doing something differently in response to this information. These results indicate areas of improvement in educational awareness about the effect of air pollution on heart disease and about related exposure-reduction behaviors, in alignment with the goals of Million Hearts.

## CRediT authorship contribution statement

**Tia C. Dowling:** Conceptualization, Methodology, Software, Formal analysis, Writing – original draft, Writing – review & editing. **Audrey F. Pennington:** Conceptualization, Writing – review & editing. **Hilary K. Wall:** Conceptualization, Writing – review & editing. **Maria C. Mirabelli:** Conceptualization, Methodology, Software, Formal analysis, Writing – review & editing, Supervision.
